# Conformational flexibility of soybean lipoxygenase is coupled to crystal solvent content in serial crystallography

**DOI:** 10.64898/2026.05.30.729005

**Published:** 2026-06-03

**Authors:** Alexander M. Wolff, Daniel W. Paley, Iris D. Young, Alexander Deary, Vidya Ganapati, Jack Hirschman, Azeem Horani, Randy Lemons, Stella Lisova, Ralph L. McAnelly, Dondis Moreland, Sandra T.M. Mous, Amanda Ohler, Joshua M. Rodriguez, Silvia Russi, Raymond G. Sierra, Daniel Mariusz Tchoń, Jan Paulo T. Zaragoza, Sergio Carbajo, Alec H. Follmer, Judith P. Klinman, Adam R. Offenbacher, Frédéric Poitevin, Nicholas K. Sauter, Mark A. Wilson, Aaron S. Brewster, Michael C. Thompson

**Affiliations:** a Department of Chemistry and Biochemistry, University of California, Merced, Merced, CA, USA; b Molecular Biophysics and Integrated Bioimaging Division, Lawrence Berkeley National Laboratory, Berkeley, CA, USA; c Linac Coherent Light Source, SLAC National Accelerator Laboratory, Menlo Park, CA, USA; d Department of Chemistry, East Carolina University, Greenville, NC, USA; e Stanford Synchrotron Radiation Lightsource, SLAC National Accelerator Laboratory, Menlo Park, CA, USA; f Department of Chemistry, University of California Berkeley, Berkeley, CA, USA; g Department of Electrical and Computer Engineering, University of California, Los Angeles, Los Angeles, CA, USA; h Department of Chemistry, University of California, Davis, Davis, CA, USA; i Department of Biochemistry and Redox Biology Center, University of Nebraska, Lincoln, NE, USA

**Keywords:** serial crystallography, protein dynamics, indexing ambiguity, polymorphism

## Abstract

Serial femtosecond crystallography (SFX) is increasingly employed to determine protein structures and study conformational dynamics under physiological conditions, but the effects of sample preparation and delivery on crystallized macromolecules remain poorly understood. Here, we report the analysis of soybean lipoxygenase-1 (SLO) microcrystals collected by SFX at the Linac Coherent Light Source. During data analysis, we observed unexpected polymorphism in SLO’s unit-cell parameters, arising from two compounding factors: indexing ambiguities caused by the pseudo-tetragonal symmetry of the SLO crystal lattice, and true non-isomorphism between individual crystals driven by differential hydration of microcrystals embedded in a hydroxyethylcellulose carrier medium. By combining unit-cell clustering with systematic reindexing, we resolved two distinct polymorphs and determined independent structures from a single experiment. Although the two structures show little difference in the average atomic coordinates (average all-atom RMSD of 0.34 Å), an approximately 8.5% difference in crystal solvent content produces measurable differences in crystal contacts and conformational flexibility. The more hydrated (large-cell) polymorph exhibits greater inter-domain flexibility and harmonic disorder in hydrophobic core residues belonging to the catalytic network of the enzyme. In the dehydrated (small-cell) polymorph, these same residues adopt discrete alternative conformations resolvable in the electron density. Our results demonstrate that sample delivery conditions in serial crystallography can significantly modulate the apparent conformational landscape of crystallized proteins, with direct implications for the interpretation of protein dynamics from SFX data.

## 1. Introduction

The role of solvent content in determining the behavior of protein crystals has been known for essentially as long as the field of macromolecular crystallography has existed. In order to collect the first ever diffraction images from protein crystals, Bernal and Crowfoot made the key discovery that their crystals only produced diffraction patterns if they remained hydrated (Bernal & Crowfoot, 1934). Later, Crick and Kendrew noted that most protein crystals have a solvent content of 40–60% (Crick & Kendrew, 1957), an observation that was roughly in agreement with a later study by Matthews relating solvent content to the packing density of protein in the asymmetric unit (Matthews, 1968). As the volume of data in the Protein Data Bank grew, Rupp and colleagues showed that there was a correlation between the solvent content of crystals and the resolution of the resulting diffraction patterns, with lower solvent content generally yielding higher resolution (Kantardjieff & Rupp, 2003; Weichenberger et al., 2015). This observation spawned numerous attempts to improve the quality of X-ray diffraction from crystals by modulating their solvent content (Bowler et al., 2006; Sanchez-Weatherby et al., 2009; Bowler et al., 2015; Russi et al., 2011; Heras & Martin, 2005). In a narrower range of contexts, the relationship between crystal solvent content and protein conformation has been explored. Notably, changes in solvent content have been associated with changes to backbone and side chain conformation, as well as changes to hydration layer structure, in lysozyme (Kodandapani et al., 1990; Madhusudan et al., 1993; Nagendra et al., 1998; Biswal et al., 2000; Atakisi et al., 2018) and bovine pancreatic ribonuclease A (Bell, 1999). The notion that protein crystal solvent content can affect the quality of X-ray diffraction data and also the conformational landscape of the crystallized proteins leads to a paradox for experimentalists. We strive for the highest possible data quality, but the sample treatments that optimize data quality could introduce artefacts into our observations.

The challenges associated with optimizing samples for high data quality come into sharp focus for serial crystallography experiments, which have gained popularity over the past decade. In a serial crystallography experiment, thousands of diffraction images comprising a dataset are collected from distinct crystal specimens, typically using batch-grown microcrystals that are no larger than tens of microns in each dimension (Chapman et al., 2011; Boutet et al., 2012; Schlichting, 2015). Sample delivery in serial crystallography often involves applying significant perturbations to crystals. For example, microfluidic jet-based systems frequently expose microcrystal samples to high pressure (Weierstall et al., 2014), high voltage (Sierra et al., 2012), or vacuum (DePonte et al., 2008). Furthermore, samples are often mixed with solutes that act as viscogens, including water-soluble polymers such as polyethylene glycol and cellulose derivatives (Kovácsová *et al.*; Sugahara et al., 2017), to alter their rheological properties and stabilize their flow. Thus, in serial crystallography, sample treatments and manipulations that are often required for sample delivery are known to modify the solvent content of crystals and the behavior of the crystallized molecules in the context of single crystal experiments (Collins et al., 2011; Atakisi et al., 2018; Heras & Martin, 2005; Hekstra et al., 2016). Compounding this situation is the fact that during serial crystallography experiments, sample parameters (e.g. viscogen concentration) and microfluidic parameters (e.g. pressure), are often manipulated “on-the-fly” to empirically optimize data collection.

In general, little is known about how perturbations associated with sample delivery impact protein crystals in serial crystallography experiments, but their effects are likely not negligible. Consequently, it is important to build an understanding of how sample delivery approaches affect the results of serial crystallography experiments broadly, and to explore this relationship for specific systems of interest. Here we report the results of a serial femtosecond crystallography (SFX) experiment on soybean lipoxygenase-1 (SLO), which highlight these considerations. SLO is an ideal system to study using SFX. It is a non-heme iron metalloenzyme (Roza & Francke, 1973; Chan, 1973; Dunham et al., 1990), so the ability of ultrafast X-ray pulses to outrun the effects of radiation damage preserves the chemistry of the active site during the structural measurements (Chapman et al., 2014). Furthermore, solvent-coupled protein dynamics have been associated with the catalytic mechanism of SLO (Zaragoza et al., 2023; Offenbacher et al., 2017; Zaragoza et al., 2019), making it an interesting candidate for time-resolved experiments, which are facilitated by the serial format of data collection (Follmer et al., 2025). When performing SFX experiments on SLO, we encountered unexpected polymorphism in SLO's unit-cell parameters. Analysis revealed the polymorphism was caused by a combination of indexing ambiguities related to the unique pseudosymmetry of the SLO crystal lattice, and true non-isomorphism caused by a change in crystal solvent content, which we believe was caused by viscogens used for sample delivery. Sorting these crystal polymorphs allowed us to determine two unique structures of SLO from a single experiment. We discovered that the two polymorphs displayed altered crystal packing, and differences in conformational flexibility. These differences were notable in the putative network connecting the protein surface to the active site, which has implications for the functional interpretation of the structures.

Our work on SLO illustrates a general challenge for the serial crystallography field: sample delivery conditions can introduce differences in protein structure and dynamics. In the case of SLO, the differences were not immediately apparent from atomic coordinates alone, and were only revealed by careful analysis of conformational flexibility. These findings are broadly relevant to the design and interpretation of serial crystallography experiments, particularly as the method is increasingly applied to study functionally important protein motions.

## 2. Results

### 2.1 SFX Data Collection from SLO Microcrystals

**Figure 1.**
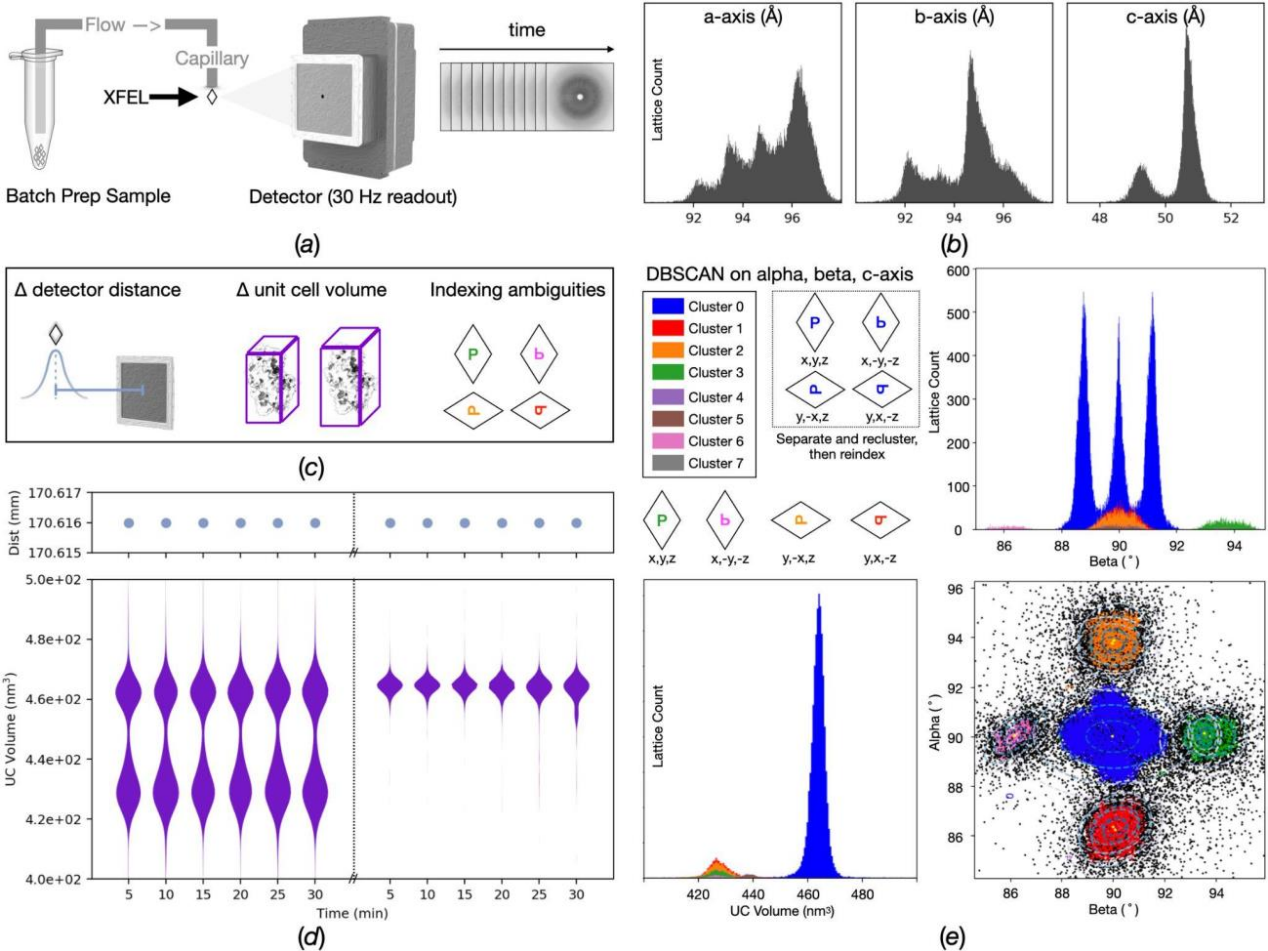
Unit cell heterogeneity in serial crystallography data collected on SLO. (a) Schematic of serial data collection highlighting batch-prepared samples which are delivered to the X-ray interaction point until a sufficient number of diffraction images are acquired for a merged dataset. (b) Histogram of unit-cell axes lengths from approximately 66,000 SLO microcrystals collected under similar conditions. (c) Illustration of potential factors driving unit cell heterogeneity. (d) Unit cell volume violin plots and detector distance scatterplot, displayed on a per-run basis where each run corresponds to ~9,000 images, collected over 5 minutes, with a sample change denoted by the dotted vertical line and corresponding break in the horizontal axis (e) Unit cell clustering analysis, with resulting clusters displayed as a function of unit cell volume, α vs β angles, and a histogram of β angles; highlighting the contributions of true unit cell volume shifts as opposed to indexing ambiguity (β angle split).

We performed serial femtosecond crystallography (SFX) on microcrystals of ferrous SLO at the Macromolecular Femtosecond Crystallography (MFX) endstation of the Linac Coherent Lightsource (LCLS). We prepared a slurry of SLO microcrystals using an established batch crystallization method (Wolff et al., 2020), starting from solution conditions that yield large, single crystals of SLO in vapor diffusion experiments (Minor et al., 1996). The batch-grown microcrystals had a rod-like morphology and were ~20–50 μm in their longest dimension. These microcrystals were concentrated and mixed with a solution of 18% hydroxyethylcellulose dissolved in crystallization mother liquor, embedding them in a gel-like medium that was delivered to the X-ray beam using a high-viscosity extruder (Figure 1A). SFX diffraction data were collected using the parameters described in Table 1.

### 2.2 Unit Cell Polymorphism Complicated by Indexing Ambiguities

During real time data analysis, after indexing approximately 66,000 lattices, we observed multi-modal distributions in each of the unit cell axis lengths (Figure 1b). Because of the dynamic nature of serial crystallography experiments and the independence of each measurement, a variety of factors can affect the unit cell parameters measured for each crystal. For example, differences in apparent unit cell dimensions can be due to changes in detector distance caused by movement of the sample position or fluctuations in the X-ray energy spectrum, as well as real polymorphism in the sample (Figure 1c), leading to variation of unit cell parameters. Batch-grown SLO crystals invariably displayed multimodal distributions for each unit cell parameter. Thus, we began by exploring whether geometry refinement (Brewster et al., 2018) could account for the observed unit cell heterogeneity. The data were split into “runs” representing bins of sequentially-collected diffraction images, with each run representing approximately 5 minutes of data collection at 30 Hz repetition rate. Examining detector distance and the distribution of observed unit cell volumes as a function of data collection run revealed a bimodal distribution in unit cell volume that was not correlated to the refined detector distance (Figure 1d). The peaks of this distribution centered around two volumes, ~460 nm^3^ and ~420 nm^3^, which we will refer to hereafter as the “large-cell” and “small-cell”. Upon switching to a new sample batch, we saw a shift in the ratio of large-cell to small-cell lattices, but both were still present. Notably, with increasing run number, and therefore time, following sample preparation, we observe the appearance of more small-cell lattices, indicating a time-dependent shift in the proportion of cell volumes.

Next, we needed to partition the large-cell and small-cell diffraction patterns for downstream processing and merging. To achieve this, we turned to clustering methods, leveraging the DBSCAN algorithm (Ester *et al.*). Upon exploring the six unit-cell parameters—*a*, *b*, *c*, α, β, and γ—it became clear that while the distribution of unit-cell volumes was bimodal, other factors were contributing to the observed unit cell heterogeneity (Figure 1e). Specifically, the distribution of *c*-axis values was bimodal, matching the distribution of unit cell volumes, yet the *a*-axis and *b*-axis distributions were more complex, indicating potential indexing ambiguity. The potential for indexing ambiguity was further supported by consideration of the expected unit cell and space group. SLO crystal structures in the PDB (e.g. 5T5V) share a common crystal form. This crystal form is monoclinic, with *P*2_1_ space group symmetry, however the *a* and *b* axes are approximately equal in length, and the β angle is nearly 90°, making the lattice pseudo-tetragonal. The consequence of this pseudosymmetry is that four different, but all valid, indexing solutions become difficult to distinguish based on unit cell parameters alone, and automated indexing algorithms struggle to apply a consistent indexing solution across all images in the dataset. In the case of SLO, if one chooses a “correct” indexing solution, then it is possible for the *a* and *b* axes to be chosen differently because they are similar in length but unique, for the direction of the c-axis to be given different polarity because the β angle is close to 90°, or for both of these mistakes to be made simultaneously.

To resolve the polymorphism and indexing ambiguity, we assigned all crystal lattices to space group *P*1, ignoring symmetry, and turned to a unit-cell clustering approach (Brewster et al., 2025). We used the α and β angles, allowing us to resolve a majority of the data into 8 clusters of interest, with a distinct 4-fold pattern. At first pass, we were able to separate and reindex 4 clusters corresponding to the small-cell, which were easier to separate due to larger deviations of the unit cell angles from 90°. We chose the cluster with α = 90° and β > 90° to be the reference, consistent with the expected monoclinic lattice, and applied one of three reindexing operators to each of the other clusters, implementing a consistent indexing solution across all images in the dataset corresponding to the small cell. On the second pass, we filtered data based on unit cell volume to isolate large-cell data, then reclustered the lattices again based on α and β. With this second set of clusters, we resolved the 4 indexing solutions for the large-cell data. Our ability to sort the data into large-cell and small-cell polymorphs and solve a two-fold indexing ambiguity for each polymorph allowed us to determine two unique structures of SLO from a single sample of batch-grown microcrystals (Tables 2 & 3).

### 2.3 Unit-Cell Polymorphism is Driven by Loss of Bulk Solvent

**Figure 2.**
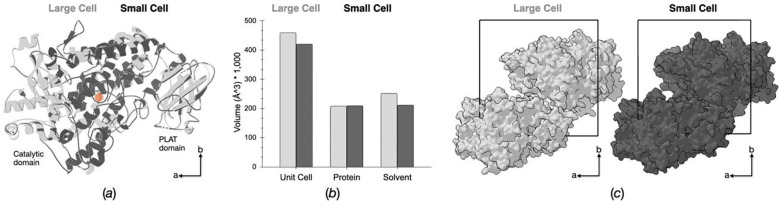
Origin of change in unit-cell volume. (a) Cartoon overlay of SLO as found in the ASU of both the large and small unit-cell structures, with the active site iron shown as an orange sphere. (b) Quantification of volume of components filling the crystallographic unit-cell for both structures. (c) Visualization of the full unit cell for each structure, with protein copies shown as surfaces while the solvent is shown as whitespace, with the cell boundaries outlined in black.

**Figure 3.**
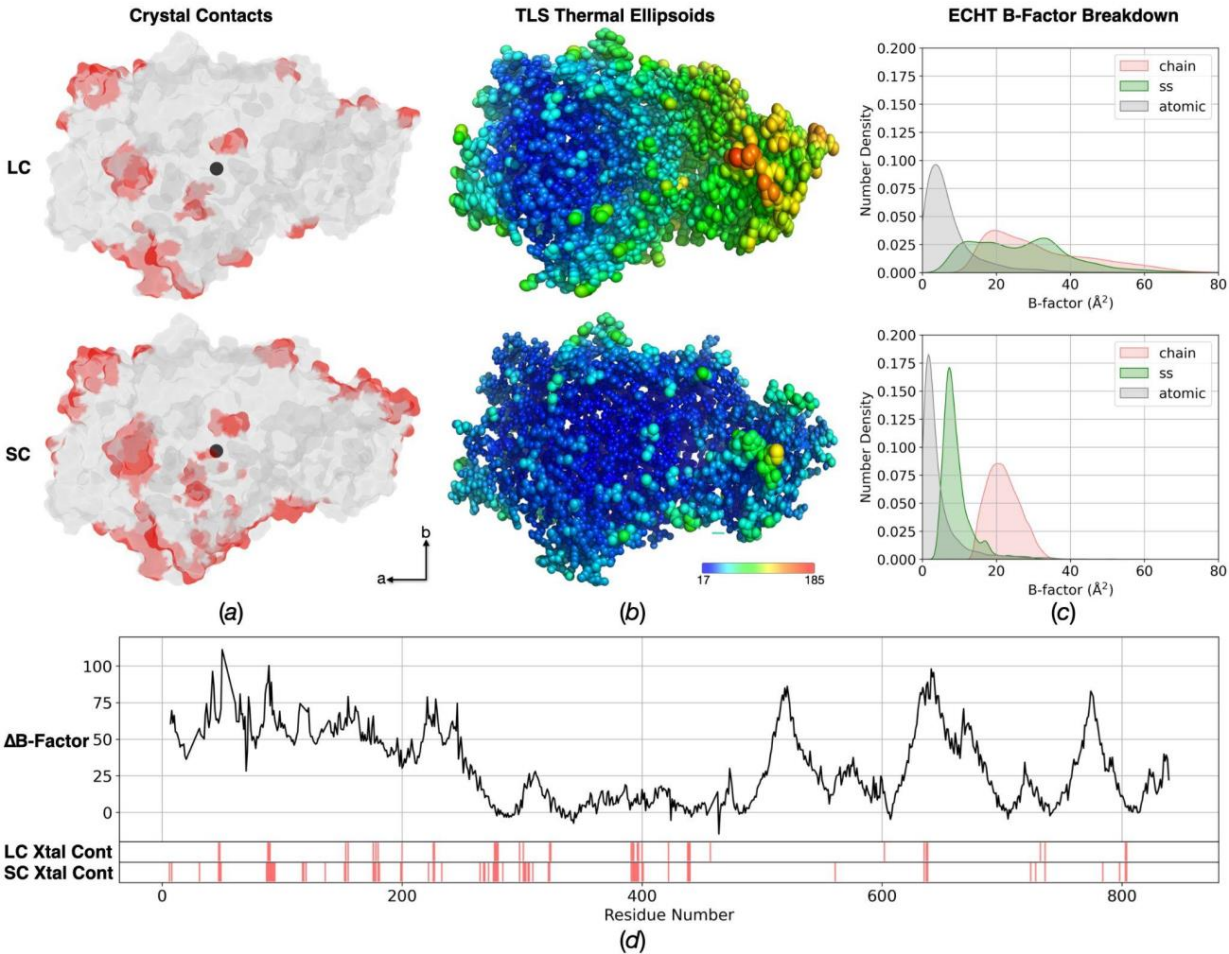
Impact of unit-cell shift on molecular motion. (a) Surface rendering of SLO with residues within 5.0 angstroms of a symmetry mate colored red and the active site iron shown as a black sphere. (b) TLS thermal ellipsoids are visualized for non-hydrogen protein atoms, with the ellipsoid's size and color scaled to the ADP's magnitude. (c) Density plot of the ADPs derived from PanDEMIC's Extensible Component Hierarchical TLS (ECHT) analysis to breakdown contributions due to chain motions, secondary structure motions, and atomic fluctuations.

After determining structures for each of the two SLO polymorphs, we sought to understand what drives the difference in unit cell volume. Given that protein crystals are composed of dynamic macromolecules and bulk water, both of which are subject to significant fluctuations, the shift in cell volume could have two potential driving forces: 1) changes in the atomic packing of SLO or 2) changes in the intermolecular spacing of molecules in the crystal lattice. These factors are interdependent, so we aimed to determine the contribution of each (Figure 2). Overlays of SLO's structure from the large-cell and small-cell data indicate similarity in overall conformation, with an average all-atom RMSD of 0.34 Å. Pairwise distance comparisons corroborate that there were no large conformational changes, yet there is evidence for small-scale movements (Figure S1). To more carefully quantify the differences, we determined that the change in volume corresponded to the large-cell losing ~8.5% of its volume (Figure 2b). In comparison, the volume of a single SLO molecule derived from the large-cell and small-cell structures was effectively equivalent, suggesting there is no significant change in the overall density of atomic packing within individual SLO molecules. After accounting for both copies in the unit cell, the protein volume remains unchanged, and the remaining volume loss can be attributed to changes in the volume of bulk solvent within the crystal. In both the large and small unit cells, SLO has roughly the same solvent-accessible surface area (SASA), indicating that the primary driving force for the loss of bulk solvent from the crystal was changes in intermolecular spacing. Visualizing both copies of the ASU and the unit cell boundaries (Figure 2c) further supported that the ~4% decrease in the a-axis and ~3% decrease in the c-axis (Table 2) were accounted for primarily by a decrease in intermolecular spacing. This observation prompted us to ask how the resulting crystal contacts were distributed and how this might alter our interpretation of SLO's structure. We mapped crystal contacts by highlighting residues within 5.0 Å of crystallographic symmetry mates (Figure 3a) for each of the two structures. The small-cell structure contained more crystal contacts, especially in the N-terminal region of SLO around the PLAT (Polycystin-1, Lipoxygenase Alpha-Toxin; residues 1–145) domain. Spatially, this change led to more extensive contacts along the direction of the crystallographic b-axis. Given that this axis had the smallest change between the cells, this indicated that molecules within the crystal may have slid and rotated within the ac plane as they packed together due to loss of solvent.

To confirm that changes in solvent content can drive changes to unit cell volume, we performed a crystal dehydration experiment. We used a large, single crystal of SLO and measured changes to the unit cell parameters as a function of relative humidity (RH) at a synchrotron beamline. We measured the unit cell volume as a function of RH and saw a trend toward larger volume at higher RH (Figure S2). Interestingly, in this humidity-dependent experiment performed on a large (approximately 50×50×200 μm^3^), single SLO crystal, the shift in volume did not reveal a sharp discontinuity as we expected based on the bimodal distribution of unit cells observed for cellulose-embedded SLO microcrystals (approximately 5×5×20 μm^3^), nor did this relationship follow a simple linear trend.

### 2.4 Differences in Crystal Packing Alter SLO's Intramolecular Dynamics

The observed shift in crystal packing from the large to the small unit cell was coupled to a decrease in overall crystallographic disorder, as evidenced by the decrease in Wilson B-factors from 32.41 to 23.15 Å^2 (Table 2) as well as the decrease in the average atomic B-factors for protein atoms from 66.11 to 35.74 Å^2 in the refined structures (Table 3). We refined our atomic models using a translation-libration-screw (TLS) model of anisotropic B-factors, and visualized the resulting thermal ellipsoids, which confirmed that TLS-modeled rigid-body rotation of SLO within the crystal lattice was significantly restricted in the small- unit cell (Figure 3b). Because the TLS model imposes strict correlations in the ADPs of assigned rigid body groups, it can absorb (and thus model incorrectly) contributions to molecular disorder that occur on length scales that are smaller than the rigid group. To separate these contributions, we performed an algebraic decomposition of the anisotropic B-factor models derived from TLS refinement (Pearce & Gros, 2021). Disorder was modelled at three levels: 1) rigid body rotations and translations of the entire protein chain 2) correlated movements of secondary structure elements and 3) contributions from individual atoms. By maximizing the contribution at each level, then modelling the remaining disorder using the next level we determined that rotations of the entire chain were insufficient to explain the refined anisotropic B-factors (Figure 3C and Figure S3). Indeed, rigid body movements of the entire protein chain within the crystal lattice differed in magnitude and direction between the two hydration states, reflecting the observed shift in crystal contacts and packing around the PLAT domain. We observed that in the large unit cell, correlated movements of secondary structure elements (ss) account for a larger contribution to the anisotropic B-factor model, which we attribute to motions between the PLAT and catalytic domains. Visually, the directionality and magnitude of the thermal ellipsoids representing the anisotropic B-factors break across the domains, and there is evidence for more intramolecular disorder in the large-cell structure with higher solvent content. The decreased atomic B-factors of PLAT domain residues in the small unit cell correlates with increased crystal contacts across this domain (Figure 3D). These trends in disorder are further corroborated by examining the location and distribution of ordered waters, and their average B-factors, which revealed increased order at the interface between the catalytic and PLAT domains in the dehydrated small-cell structure (Figure S4).

### 2.5 Active-site Geometry is Stable Despite Perturbations to Catalytically Coupled Residues

**Figure 4.**
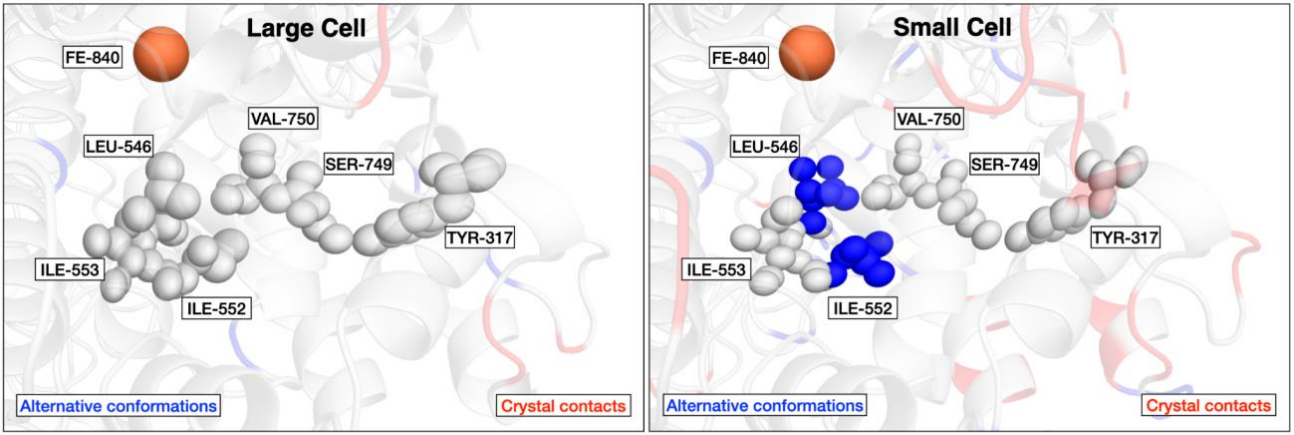
Unit-cell shift affects SLO’s thermal network. Cartoon renderings of the large and small cell SLO structures, with alternative conformations highlighted in blue and crystal contacts highlighted in red. The active-site iron is shown as an orange sphere, and thermal ellipsoids are illustrated for select residues previously demonstrated to be part of a thermal network coupled to the rate-limiting step of catalysis.

Changes in atomic B-factors reveal alterations to a protein’s conformational ensemble that can be modeled using a harmonic approximation, and we were also curious whether the two unit cells would manifest differences in features of the protein structure that are modeled anharmonically. To investigate this question we asked which residues had evidence for alternative conformations in each of the two unit cell settings (Figure S5). In the small cell, SLO has more surface residues displaying alternative conformations. Many of these residues are part of newly formed crystal contacts in the small-cell structure, highlighting how crystal packing can stabilize alternative side chain conformations at an interface. Internal residues also display changes in how their side chain conformational heterogeneity is modeled crystallographically (Figure 4). Specifically, we found that the ideal method for modeling side chain conformational heterogeneity in residues LEU546 and ILE552, both buried hydrophobic residues, differs for the small and large unit cell structures. In the small-cell structure, each of these residues populates two rotameric states that are evident in the electron density, can be modeled discreetly, and their occupancies refined (Figure S6). In the large-cell structure, only a single rotamer is evident in the electron density, the density is more diffuse, and the refined B-factor for the side chain atoms is higher, suggesting the underlying heterogeneity is best modeled harmonically, rather than with alternative conformations. The difference in the interpretation of conformational heterogeneity is notable, given that the same resolution cutoff was applied to each data set and the data quality indicators are similar for the two data sets (Table 2). In contrast to the differences noted for L546 and I552, both the electron density and refined model corresponding to the non-heme iron in the enzyme active site appear unchanged by changes in hydration and crystal packing (Figure S6). The measured distances between the iron atom and the residues which coordinate it are nearly identical in the two structures.

## 3. Discussion

In this work, we examined SLO microcrystals using SFX, and found that our samples contained a mixture of two polymorphs, allowing us to determine two unique crystal structures with the same space group symmetry, but differently sized unit cells. Analysis of the diffraction data was complicated by the presence of pseudosymmetry, caused by the *a* and *b* axes of the monoclinic unit cell being nearly identical in length, and the *β* angle being almost exactly 90॰. We sorted the crystal polymorphs based on their unit cell volumes, and applied a uniform indexing solution to the individual lattices based on a clustering approach (Brewster et al., 2025). The main difference between polymorphs is a difference in solvent content, but otherwise the resulting protein structures were nearly identical in terms of their atomic coordinates, with an average all-atom RMSD of 0.34 Å. However, TLS refinement showed that the subtle change in crystal packing between polymorphs affects the dynamics of SLO in the crystal. Furthermore, the interpretation of conformational heterogeneity in the core of the protein based on the electron density maps differs between polymorphs. This study highlights the interplay between experimental and analytical challenges in serial crystallography.

Our data illustrate two consistent complications that are inherent to serial measurements: crystal pseudosymmetry and sample polymorphism. Issues arising from pseudosymmetry, where the crystal lattice has higher symmetry than the true space group, were appreciated early on in the development of serial crystallography, and led to the development of clustering algorithms to unravel the “computational twinning” that would arise from applying an inconsistent indexing solution to individual lattices within the data set (Brehm & Diederichs, 2014; Gildea & Winter, 2018). Furthermore, other serial crystallography studies have also reported unit cell polymorphism that is similar to (and in some cases, even more complex than) what we observe in our data (Ebrahim et al., 2018; Stagno et al., 2017; Brewster et al., 2025). As serial crystallography becomes increasingly common, it is becoming apparent that these challenges are not rare edge cases, but frequent problems faced by practitioners. In our case, the presence of two very clear peaks in a histogram of unit cell volumes revealed the existence of two specific polymorphs, and allowed efficient sorting of the lattices into two groups. Observing the symmetry of *α* and *β* angle distributions in our data (Fig. 1e) suggested that there could be an indexing ambiguity, and consideration of the expected unit cell axis lengths and angles confirmed the possibility of pseudosymmetry and allowed us to determine a suitable set of reindexing operators. After developing an appropriate workflow for data processing that included sorting and reindexing, we were able to determine structures for each polymorph.

We investigated the atomic models refined against the large and small cell data sets, and compared them. Our initial investigation revealed negligible changes to the overall structure of SLO, with the main difference between the two polymorphs being that the smaller, more compact unit cell has less solvent and more extensive crystal contacts between neighboring SLO molecules. The observation that the distribution of polymorphs changes as a function of time following mixing into the hydroxyethyl cellulose carrier medium (Fig. 1d) supports the notion that polymorphism is a consequence of exposure to the carrier medium. The bimodal distribution of polymorphs in our microcrystal sample is consistent with a first-order phase transition driven by expulsion of solvent from the crystal lattice. Similar discontinuous phase transitions in macromolecular crystals involving changes in crystal solvent content as a function of water vapor pressure have been reported for hemoglobin (Huxley & Kendrew, 1953) and lysozyme (Dobrianov et al., 2001). It is unclear why our single crystal measurements did not reveal a sharp discontinuity in unit cell dimensions as a function of relative humidity (Fig. S2). Dobrianov, et al. noted that crystal size and shape can play a role in equilibration times for phase transitions in macromolecular crystals, and that these transitions can occur inhomogeneously (Dobrianov et al., 2001), which could explain why we observe complex behavior from a large single crystal, and a clear, first-order phase transition for microcrystals. Previous work in serial crystallography also supports the notion that small crystals are more robust to changes in unit cell dimensions induced by experimental perturbations (Stagno et al., 2017).

Although our initial inspection of atomic coordinates showed little difference between the small and large cell SLO models, an analysis of anisotropic B-factors refined using TLS parameters revealed that the changes in crystal packing and solvent content have considerable effects on the conformational flexibility of SLO molecules in the crystal lattice. We observed increased B-factors in the PLAT domain of the large cell model, which cannot be explained by rigid body displacements of the crystallized molecules alone. Instead, anisotropic B-factors suggest that the crystal packing in the large cell is more permissive of collective motions that alter the relative positioning of the catalytic and PLAT domains, comparable to the rocking motion suggested by solution scattering measurements on SLO (Dainese et al., 2005) and homologous lipoxygenases (Hammel et al., 2004). In addition to these differences in domain scale dynamics, we also noted an interesting difference in the manifestation of conformational heterogeneity in the core of the protein, exemplified by residues L546 and I552, which are both of functional significance (Offenbacher et al., 2017; Zaragoza et al., 2023). In the large cell, electron density maps reveal only a single side chain conformation, but the refined B-factors suggest significant heterogeneity for these sidechains. In the small cell, two distinct conformations can be modeled in the electron density, each with lower B-factors than the single conformations modeled in the large cell. This observation raises the question of whether the crystal packing changes the nature of the enzyme’s dynamics, with the larger unit cell promoting harmonic movements of these sidechains and the small cell promoting anharmonic motions involving exchange between rotameric wells, or whether the overall increased motion in the large cell leads to a blurring of the electron density that simply eliminates our ability to resolve the alternative conformations of these sidechains. These apparent differences in the electron density exist, despite the fact that the two structures were refined against diffraction data with a similar resolution cutoff. Along the same lines, the small cell model has significantly more ordered water molecules than the large cell model, despite being of a similar resolution. We likewise cannot determine whether this is because the enzyme interacts with fewer water molecules in the more expanded crystal lattice, or whether our ability to resolve them is compromised by increased flexibility of the protein.

In our efforts to establish effective sample delivery conditions for our SFX experiments, we overlooked the potential effect of viscogens inducing loss of solvent content and polymorphism in our SLO microcrystals, a common blindspot in the SFX field. In hindsight, other reports, including some of our own work, show that such viscogens can alter the dimensions of the crystal lattice. For example, CypA microcrystals measured in viscous hydroxyethyl cellulose show a 2% reduction in volume relative to the same crystals measured in a sample delivery medium lacking the viscogen (Wolff et al., 2020). Additionally, SFX experiments on photosystem II revealed that increased PEG concentrations lead to a 6% decrease in unit cell volume, which resulted in significantly lower Wilson B-factors and increased resolution (Young et al., 2016). In the case of our SLO crystals, the putative effect of altering the crystal solution conditions is not uniform, resulting in the observed crystal polymorphism. At first, the difference between the two polymorphs present in our microcrystal samples seemed inconsequential - despite a difference in solvent content, the crystal resolution and data quality were comparable, and the refined atomic coordinates were essentially identical. A more thorough inspection revealed important differences in the manifestation, and possibly on the interpretation, of the enzyme’s internal dynamics in the different crystal forms. Because SFX measurements are frequently employed in time-resolved crystallography experiments that aim to explore the dynamics of the crystallized molecules, our study underscores the importance of considering how the functional motions of proteins can be altered by crystallization and sample delivery conditions.

## 4. Materials and Methods

### 4.1 Soybean lipoxygenase expression and purification

SLO was expressed and purified as previously described (Hu et al., 2014; Offenbacher et al., 2017). Briefly, the plasmid containing an untagged, wild-type SLO gene was transformed into BL21(DE3) Codon Plus RIL cells. Improved expression yields were found when the overnight LB starter was supplemented with ampicillin and 0.4% glucose and grown overnight with shaking at 30 °C (Jakobowski et al., 2024). The overnight was added to 2×YT media (100-fold) containing AMP and 3% ethanol, and grown at 37 °C to an OD600 of 1.0–1.2. The temperature of the shaker was reduced to 18 °C and the cells were collected after 48 hours. The cells were resuspended in a lysis buffer and sonicated. The SLO protein was exchanged in 20 mM Bis-Tris, pH 6.0 and isolated in two steps with two cation exchange columns (SP Sepharose and UNO S6 [Bio-Rad]). For each column, the sample was loaded and washed with 2 column volumes of 20 mM Bis-Tris,pH 6, followed by elution with a linear gradient (0–500 mM NaCl in 20 mM Bis-Tris buffer, pH 6). The eluted protein was dialyzed in 0.1 M borate, pH 9 buffer and stored at −80 °C until further use. The protein, as isolated, was estimated to contain approximately 0.8 iron atoms per protein in the ferrous state.

For crystallography, additional purification steps were performed. SLO (from above) was thawed and dialyzed for three hours in 50 mM sodium acetate (pH 5). Aggregation was removed by centrifugation and the protein was further purified with a HiPrepTM 26/60 SephacrylTM S-200 HR column equilibrated in 50 mM HEPES, 150 mM NaCl (pH 7.4). Protein samples were stored at −80°C until further use. Upon thawing, protein was immediately dialyzed back into 50mM sodium acetate (pH 5) prior to crystallization.

### 4.2 Seed stock preparation

SLO at 5 mg/mL in 50 mM NaOAc, pH=5.0, was mixed in a 1:1 ratio with mother liquor (0.4M NaOAc pH=5.5, 8–10% PEG-3350) in 2 μL hanging drops. Crystals formed within 5–7 days (Steczko et al., 1990). Crystals from a single hanging drop were crushed, then diluted to ~50 uL with mother liquor. This seed stock was used to make the first batch of microcrystals. Subsequent seed stocks were prepared by taking ~10μL from previous batch crystal solutions, crushing the crystals, then diluting with mother liquor to ~50 μL.

### 4.3 Batch Crystallization

SLO at 10 mg/mL in 50 mM NaOAc, pH=5.0, was mixed in a 1:1 ratio with batch precipitant (0.4M NaOAc pH=5.5, 16% PEG-3350) to a volume of 1 mL in a 1.5 mL Eppendorf Tube. ~10μL of seed stock (see 2.2.1.) was added to the tube cap. The tube was then placed on a Thermo Fisher Tube Revolver Rotator, set to 30 RPM. Samples were left to mix overnight. When ready for use, crystals were allowed to settle at the bottom of the Eppendorf Tube, and excess crystallization buffer was removed. Crystals were mixed in a ~1:1 ratio with 18% (w/v) hydroxyethylcellulose (Sigma-Aldrich PN-09368) dissolved in crystallization buffer (0.225M NaOAc pH=~5.43, 8% PEG-3350). Each component was loaded into a Hamilton Syringe, and both syringes, along with a third empty syringe, were connected to a custom 3-way coupling device (James et al.). The batch crystals and cellulose were mixed together and immediately deposited in reservoirs for use with the HVE (high viscosity extrusion) injector (Weierstall et al., 2014).

### 4.4 Serial crystallography data collection

SFX data were collected at the MFX endstation of LCLS using the Rayonix MX340-HS detector in 2×2 binning mode. Crystals were delivered to the X-ray interaction point using an HVE injector, and data were collected using a ~5 μm beam at ~9.5 keV delivered at 30 Hz, with a pulse duration of ~30 fs. An energy scan was collected across a range of +/− 0.05 keV while monitoring the energy spectra using a crystal transmissive spectrometer (Zhu et al., 2013) upline at the XPP instrument (Chollet et al., 2015), which allowed for per-shot energy calibration. Detector geometry was calibrated using silver behenate powder diffraction patterns. Further parameters are provided in Table 1.

### 4.5 Diffraction data processing

Data collection was monitored and processed using the *cctbx.xfel* software suite, based on DIALS and CCTBX (Sauter et al., 2013; Brewster et al., 2025). Spot-finding and initial indexing were carried out using *dials.stills_process*. Images were indexed in space group *P*1 using a target unit cell of (96.03, 94.55, 50.54, 90, 91.15, 90). Initial indexing results were clustered using the *uc_metrics.dbscan* command, which leverages the DBSCAN algorithm (Ester et al.) to cluster cells based on values for α, β, and the c-axis. The hyperparameter ε, representing a length scale, was optimized to ensure maximum capture of cells while prioritizing separation of clusters. For a given set of cells assigned to a cluster, reindexing operators were applied to ensure that the mean value of β was >90°. Symmetry constraints appropriate to space group P12_1_1 were then applied, forcing α and ɣ to 90°. Clusters with common c-axis lengths (corresponding to a common unit-cell volume) were then subjected to the Brehm-Diederichs method (Brehm & Diederichs, 2013) to break polar ambiguities using the *modify_cosym* (Gildea & Winter, 2018) module within *cctbx.xfel.merge* (Brewster et al., 2025). Finally, these data were merged and postrefined, with error estimates treated using the Ev11 method (Brewster et al., 2018; Evans, 2011). Thus, from one stream of data two independent datasets with different unit cell volumes were produced.

### 4.6 Structural modeling

Intensities as output from *cctbx.xfel.merge* were phased via molecular replacement using Phaser (McCoy et al., 2007), with chain A from PDB entry 5t5v as a search model. R-free flags were generated and random atomic displacements (0.5 Å) were applied to minimize model bias prior to initial refinement. The model was refined in PHENIX (Afonine et al., 2012), beginning with a rigid-body strategy. This was followed by manual rebuilding using Coot (Emsley & Cowtan, 2004) and subsequent refinement in PHENIX. This iteration continued until the model converged. The models and structure factors were deposited to the PDB under codes 9O4S & 9O4T. Statistics for refinement are provided in Table 3. TLS refinement was applied to both models, with 1 TLS group used for the small-cell structure and 3 TLS groups used for the large-cell structure. Contributions to TLS ellipsoids were separated using ECHT (Pearce & Gros, 2021), restricting the model to chain, secondary structure, and atomic levels (auto_levels=chain+ss) with the remaining parameters set to default values.

### 4.7 Dehydration Experiments

Crystals were grown as described above, adjusting protein concentration from 15 mg/mL down to 4–6 mg/mL (0.2M NaOAc pH=5.5, 9% PEG-3350) to ensure growth of larger crystals. Individual crystals were harvested in a MiTeGen SLEEC humidity-controlled chamber and stored overnight in custom cassettes from MiTeGen. Crystals were mounted open to air under a humidity-controlled jet (Sanchez-Weatherby et al., 2009) at SSRL BL12–2. Relative humidity was set to 99.5%, a series of 10 images were collected using 0.5 ° oscillations for a total exposure of 50 kGy. The crystal was translated to avoid radiation damage, rotated to the original phi angle, humidity was adjusted to 98.5%, 5 minutes for equilibration was given, then 10 images were collected as above. This procedure was repeated for a descending range of relative humidities until the crystal no longer diffracted well. Data were indexed using *dials* and *Xia2*, and the corresponding unit-cell volume was calculated for each humidity setpoint.

## Supplementary Material

Supplement 1

## Data Availability

Structural models and associated reflection data are deposited in the PDB under accession codes 9O4S & 9O4T. The model used for molecular replacement is available under code 4WFO. Raw diffraction frames are deposited in CXIDB under 242.

## Synopsis

Using soybean lipoxygenase-1 (SLO) as a model system, we show that the carrier media used for sample delivery in SFX experiments can alter the solvent content of protein microcrystals, producing distinct crystal polymorphs within a single experimental sample. Although the average atomic coordinates are minimally perturbed, a detailed analysis of crystallographic displacement parameters reveals that conformational flexibility is sensitive to sample delivery conditions, a consideration of broad relevance for time-resolved SFX experiments that aim to capture functionally important protein motions.
