# Supplementary material for: Conformational flexibility of soybean lipoxygenase is coupled to crystal solvent content in serial crystallography
Source: bioRxiv. 2026 Jun 3:2026.05.30.729005. Preprint. [Version 1] doi: 10.64898/2026.05.30.729005 (PMC13252045; doi:10.64898/2026.05.30.729005)
Supplement: Supplement 1 [file media-1.pdf]

Supporting Information for

Conformational flexibility of soybean lipoxygenase is coupled to crystal solvent content in serial crystallography

Alexander M. Wolff, et al.

Table 1. Sample delivery and X-ray diffraction parameters for data collection.

Delivery Matrix	18% hydroxyethyl cellulose
Sample flow rate (μL/min)	0.5
Capillary diameter (μm)	75
Linear jet velocity (mm/s)	1.89
Pressure (psi)	~3200
X-ray Source	LCLS - MFX
Photon Energy (keV)	9.5
X-ray pulse duration (fs)	~30
Photons per pulse	~1*10^{12}
X-ray Repetition Rate (Hz)	30

Table 2. Crystallographic statistics[a]

No. of images	442472
Hits	65813 → 14.67%
Indexed	56902 → 86.46%

Dataset	Small Cell	Large Cell
Lattices Reindexed	10181	34631
Resolution Range	19.93-1.95 (1.98-1.95)	19.59-1.95 (1.98-1.95)
Space Group	$P12_11$	$P12_11$
Unit-cell params		
a (Å)	92.04+/-1.12	95.97+/-0.31
b (Å)	93.08+/-0.96	94.56+/-0.23
c (Å)	49.03+/-0.23	50.53+/-0.10
α (º)	90	90
β (º)	92.73	91.17
γ (º)	90	90
Total Reflections	5656883 (11330)	5099225 (39552)
Multiplicity	94.00 (3.77)	77.59 (12.19)
Completeness (%)	99.06 (87.53)	99.88 (99.78)
Mean I/sigma (I)	5.068 (0.622)	4.416 (0.911)
Wilson B (Å^2)	23.15	32.41
R-split (%)	12.1 (80.5)	10.9 (61.8)
CC-int (%)	99.1 (47.1)	98.9 (56.7)

[a]Statistics for the highest-resolution shell are shown in parentheses.

Table 3. Model refinement statistics[a]

Dataset	Small Cell	Large Cell
PDB ID	9O4S	9O4T
Resolution range	19.92 - 1.949 (1.99 - 1.95)	19.59 - 1.95 (2 - 1.95)
Reflections used in refinement	59569 (3476)	65618 (4590)
Reflections used for R-free	2129 (126)	2012 (137)
R-work	0.1690 (0.3630)	0.2101 (0.3120)
R-free	0.2106 (0.3912)	0.2346 (0.3545)
Number of non-hydrogen atoms	7244	6772
macromolecules	6774	6501
ligands	1	1
solvent	469	270
Protein residues	818	813
RMS(bonds)	0.003	0.002
RMS(angles)	0.67	0.59
Ramachandran favored (%)	96.55	96.13
Ramachandran allowed (%)	3.45	3.50
Ramachandran outliers (%)	0.00	0.37
Rotamer outliers (%)	0.79	1.31
Clashscore	1.55	2.59
Average B-factor	36.03	65.70
macromolecules	35.74	66.11
ligands	23.32	43.02
solvent	40.15	55.78
Number of TLS groups	1	3

[a]Statistics for the highest-resolution shell are shown in parentheses.

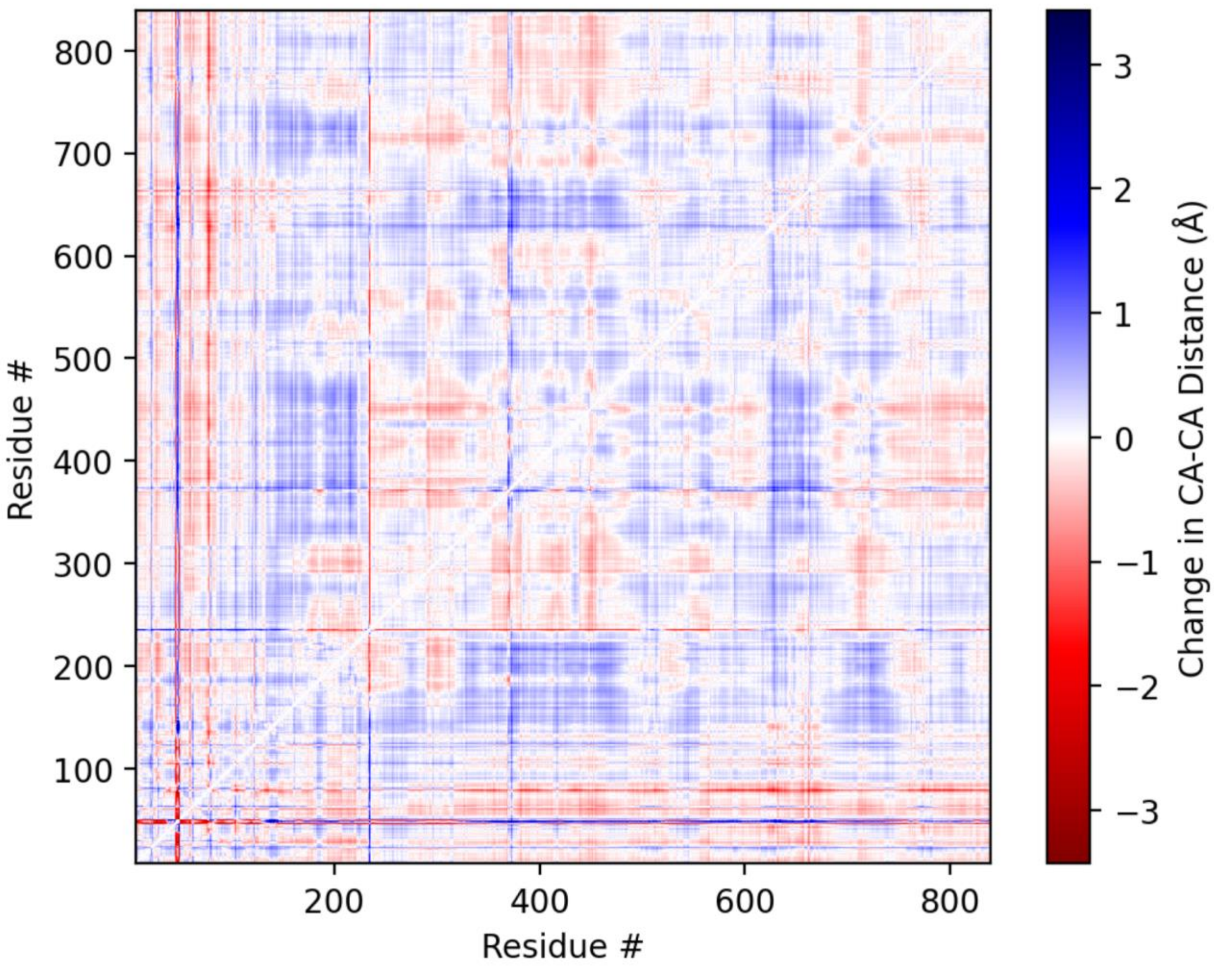

Figure S1. Overview of structural changes in large-cell to small-cell structure. A heatmap showing the internal CA-CA distance matrix of the small-cell structure subtracted from the internal CA-CA distance matrix of the large-cell structure reveals patterns of structural variation greater than the average RMSD of 0.34 Å would imply.

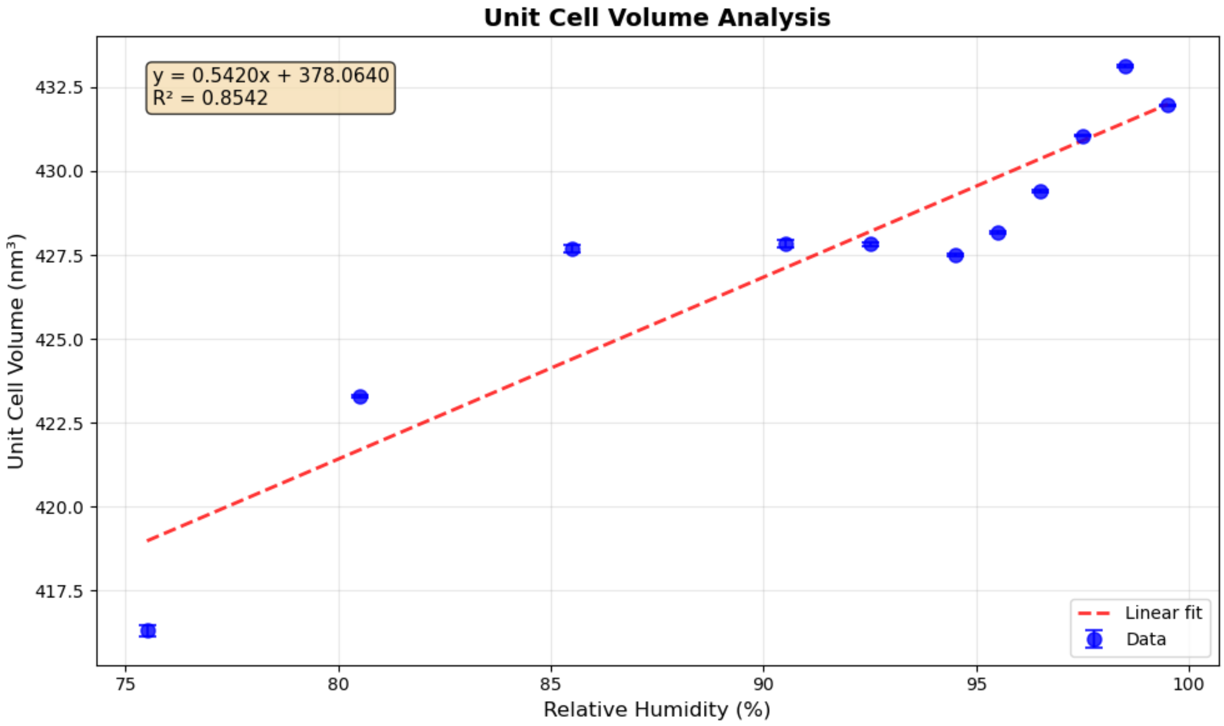

Figure S2. Hydration dependent changes in unit-cell volume of a single SLO crystal. Calculated unit-cell volume is shown based on indexing results from a series of 5-degree wedges collected from a single SLO crystal. Hydration was controlled using a humidity jet, beginning at 99.5% RH, following a change in the RH setting the crystal was given 5 minutes to equilibrate before collecting the subsequent wedge of images to assess the unit-cell volume.

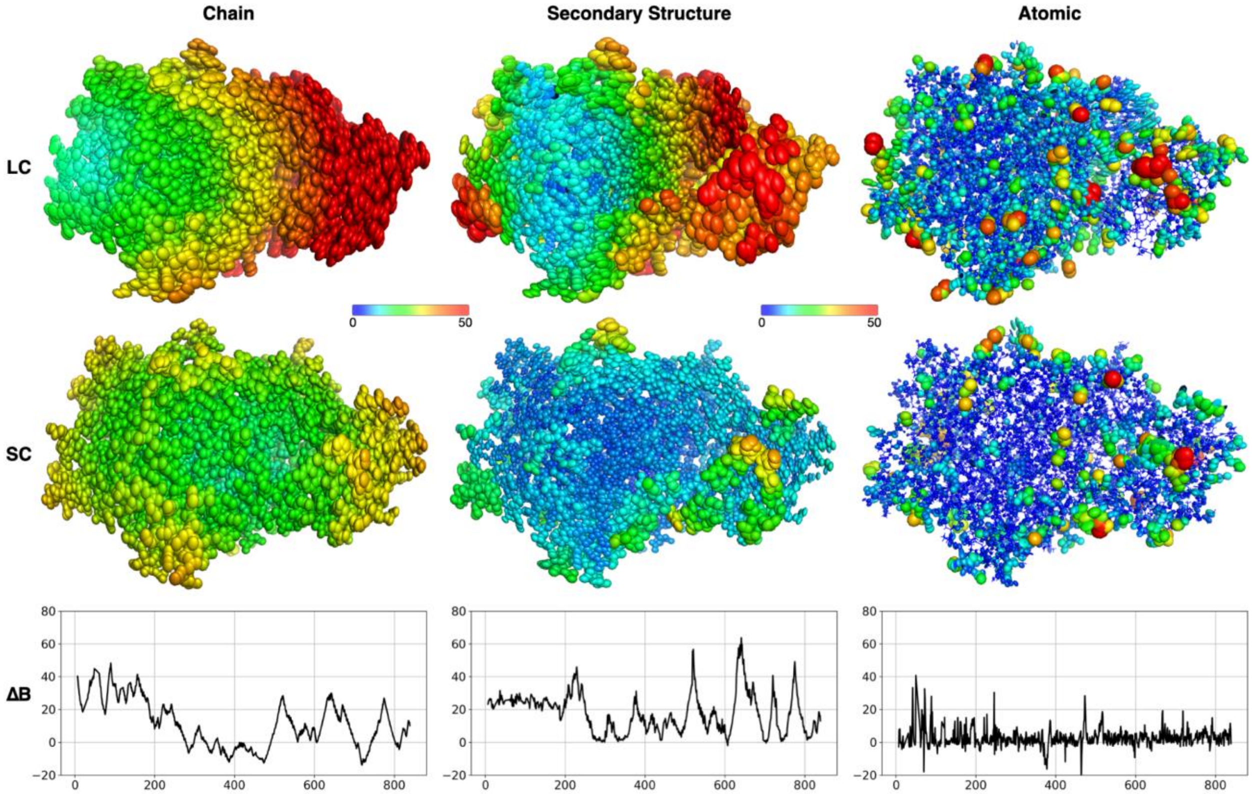

Figure S3. ECHT B-Factor Differences Between Hydration States. TLS thermal ellipsoids are visualized for non-hydrogen protein atoms, with the ellipsoid's size and color scaled to the ADP's magnitude. ADPs from the full model were separated using PanDEMIC to break down contributions due to chain motions, secondary structure motions, and atomic fluctuations, with each component visualized independently. In the bottom row the difference between the B factors (large cell - small cell) is plotted on a per-residue basis.

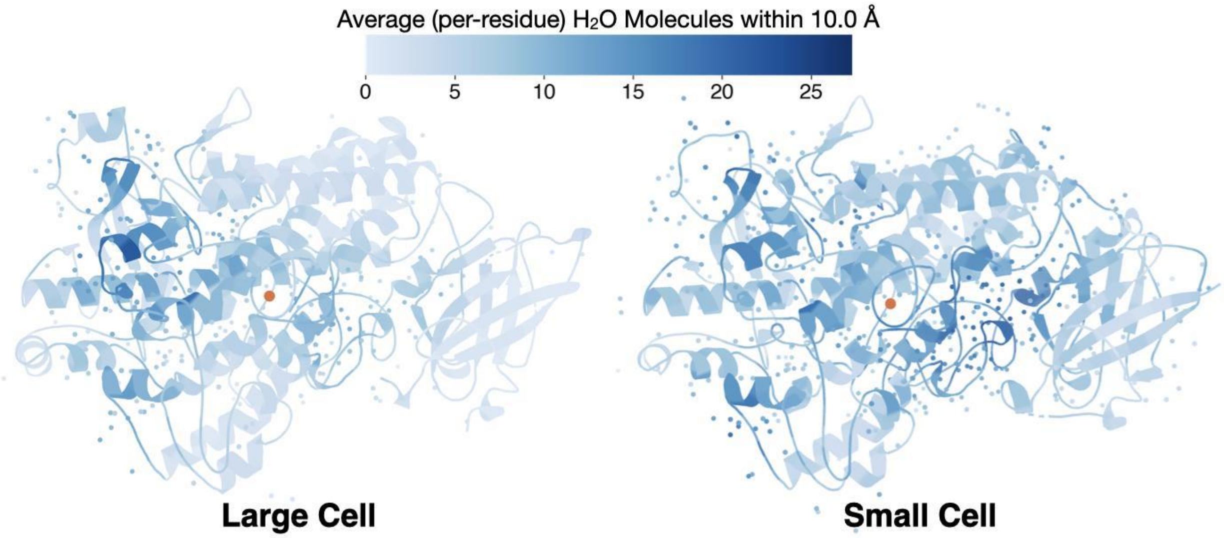

Figure S4. Distribution of Ordered Waters. SLO's structure is illustrated as a cartoon, with the active site iron shown as an orange sphere and ordered waters shown as smaller spheres. The cartoon and ordered waters are colored based on the average count of H_20 molecules within a 10 Å radius of the residue, highlighting that there are more ordered waters in the small cell structure, which are also distributed differently across the molecule.

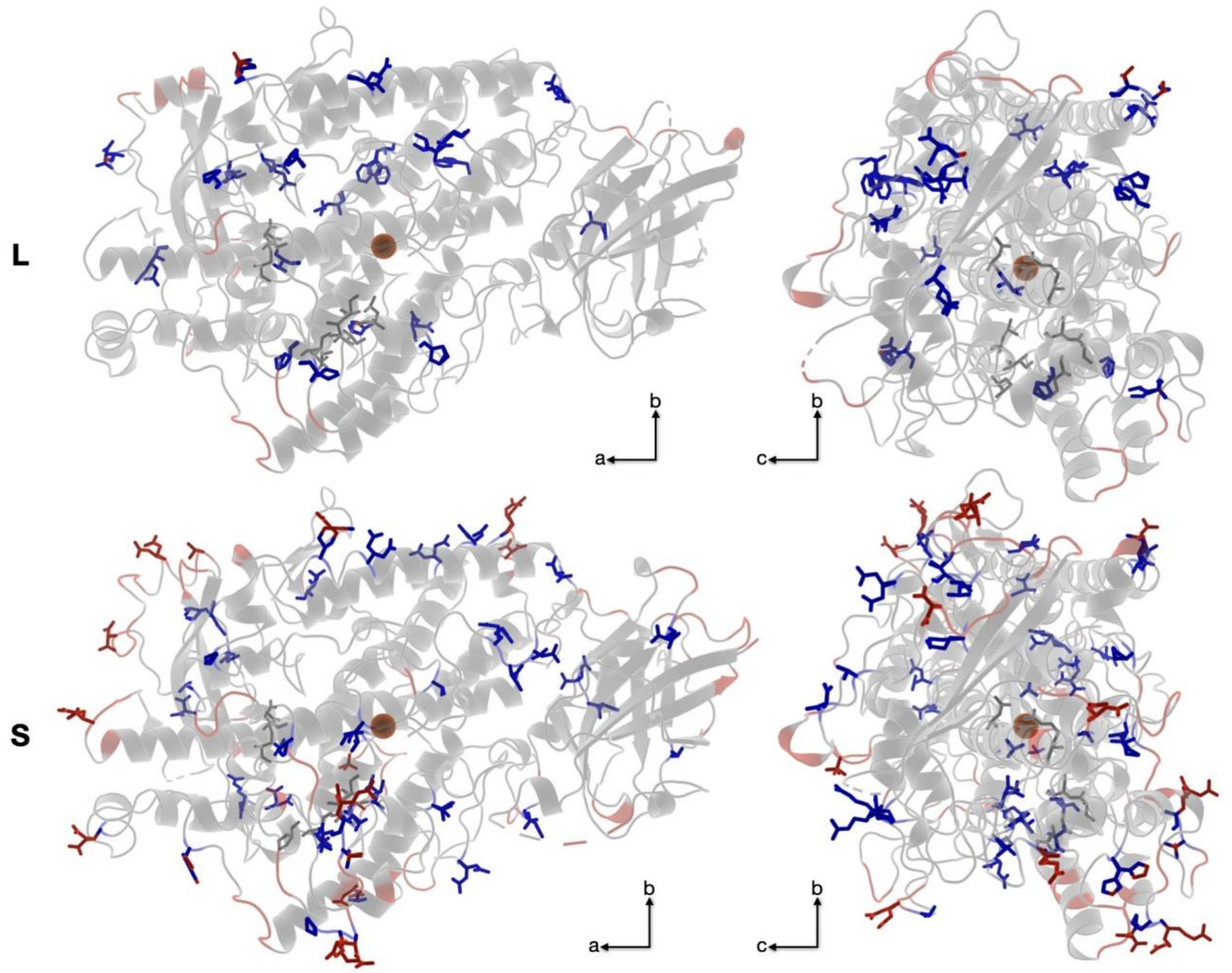

Figure S5. Distribution of Alternative Conformations. SLO's structure is illustrated as a cartoon, for the large cell (top panels) and small cell (bottom panels), with the active site iron shown as an orange sphere and residues with alternative conformations modeled shown as blue or red sticks. Blue denotes alternative conformations only, while red denotes crystal contacts. There are a greater number of alt confs in the small cell structure, with regional disparities focused near the active site.

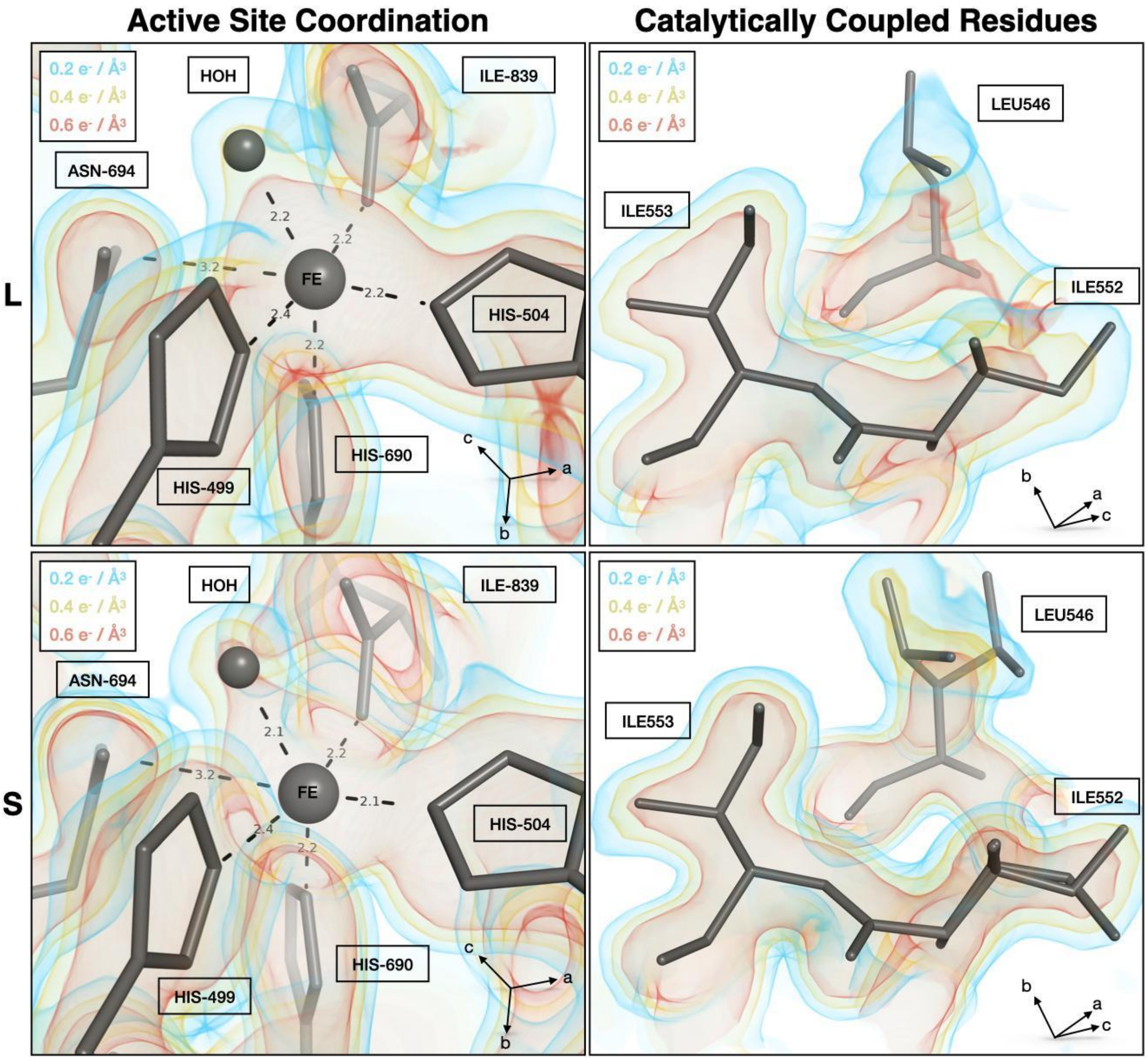

Figure S6. Iron coordination is consistent yet adjacent residues are perturbed. Illustration of the active site iron (left panels) and catalytically coupled residues (right panels) in the large cell (top panels) and small cell (bottom panels) structures. Atoms are are depicted as sticks or spheres and the 2mFo-DFc maps are rendered as volumes carved within 2.0 Å of the model, visualized at 0.2 $e^-/Å^3$ (blue), 0.4 $e^-/Å^3$ (yellow) and 0.6 $e^-/Å^3$ (red).
